# Serres’ Address on Opening the Medical Congress of Paris

**Published:** 1846-02

**Authors:** 


*M. Serres' Address on opening the Medical Congress of Paris.*—The deliberations of the congress were opened by a short, but most energetic address from M. Serres, the president, of which the following is a summary:

"Gentlemen, and dear colleagues,—Social epochs present characters which distinguish them, and stamp their impress upon them. The ruling character of the nineteenth century is, the tendency to perfecting the physical and moral welfare of man. Sciences, arts, private and general industry, every thing tends towards this end; every one is carried along by this general movement, men as well as governments. Among human sciences, there is one which, for the last three thousand years, investigates the wants of humanity, and ever meditates upon them. To this science humanity owes, in a great measure, the physical welfare which it now enjoys. This science is medicine, the dominion of which embraces surgery, pharmacy, and the veterinary art. Thus it is that the medical family folds, as it were, within its arms, society in general. It ploughs the waves with our vessels. It accompanies our soldiers in the camp, and on the field of battle. It watches at the domestic fireside, in hospitals, and in prisons. Human vicissitudes meet it nearly everywhere, and everywhere they find it prepared to make the greatest sacrifice. The more important, the more indispensable, the services which the medical body renders to society, the more attentive a government should be to the institutions which regulate it. Thence the anxiety of our government to improve the laws which govern medical institutions. It is our duty to acquire as much information as possible, in order that we may be of the greatest possible utility to our fellow-creatures. It is the duty of government to protect us in the application of the knowledge we have thus acquired, and to see that all the members of the social body benefit by it. One of our great men has said, 'I know nothing in the world more contemptible than a cowardly soldier, or than an ignorant medical man; the first is contemptible, because he compromises the fate of his country in the day of danger; the second, because he compromises, hourly, the life and welfare of his fellow-creatures.' Thanks be to heaven, there is not a cowardly soldier in France, and government is under the obligation to society to employ all its power to render it *impossible* that there should be such a person as an ignorant medical practitioner.—*London Lancet.*

